# The Association Between Abnormal Vital Signs and Mortality in the Emergency Department

**DOI:** 10.7759/cureus.20454

**Published:** 2021-12-16

**Authors:** Jood H Simbawa, Abdulkarim A Jawhari, Fay Almutairi, Ahlam Almahmoudi, Bashair Alshammrani, Raneem Qashqari, Ibtihal Alattas

**Affiliations:** 1 Medicine, King Abdulaziz University Hospital, Jeddah, SAU; 2 Faculty of Medicine, King Abdulaziz University Hospital, Jeddah, SAU; 3 Medicine and Surgery, King Abdulaziz University Faculty of Medicine, Jeddah, SAU; 4 Emergency Medicine, King Abdulaziz University Faculty of Medicine, Jeddah, SAU

**Keywords:** ctas, heart rate, emergency department, mortality rates, vital signs

## Abstract

Background

The emergency department (ED) receives patients from all over the world every day. Hence, using various triage scales to detect sick patients and the need for early admission are essential. Triage is a process used in the ED to prioritize patients requiring the most urgent care over those with minor injuries based on medical urgency and medical needs. These decisions may be based on patients’ chief complaints at the time of their ED visit and their vital signs. Vital signs, including blood pressure (BP), respiratory rate (RR), heart rate (HR), and body temperature, are necessary tools that are traditionally used in the ED during procedures such as triage and recognizing high-risk hospital inpatients. This study aimed to determine the relationship between abnormal vital signs and mortality in the ED.

Method and Material

This retrospective record review study was performed at the ED of King Abdulaziz University Hospital (KAUH). Altogether, 641 patients fulfilled our inclusion criteria. Data including patients’ demographics, vital signs, in-hospital mortality, triage level, and precipitating factors were collected.

Results

The mean age of the patients was 45.66 ± 18.43 years (69.3% females), and the majority of them had Canadian Triage and Acuity Scale (CTAS) level 3 (71.1%). The total number of in-hospital mortalities was 32 (5%). Lower systolic blood pressure (SBP) and diastolic blood pressure (DBP), high respiratory rates, and low oxygen saturation (O_2_SAT) were significantly associated with high mortality rates.

Conclusion

Abnormal vital signs play a major role in determining patient prognosis and outcomes. Triage score systems should be adjusted and carefully studied in each center according to its population.

## Introduction

The emergency department (ED) has a highly stressful environment with a large number of sick patients [1]. Due to a large number of patient visits, quick evaluation of each patient by an emergency doctor is nearly impossible [2]. Hence, the use of various triage scales to detect medically ill patients who require quick and early evaluation and admission is mandatory in the ED [3]. Triage is a process used in the ED to prioritize patients requiring the most urgent care over those with minor injuries based on their medical urgency and medical needs [3-6]. These decisions are usually based on patients’ vital signs and chief complaints at the time of their ED visit [7].

Vital signs such as heart rate (HR), blood pressure (BP), respiratory rate (RR), and body temperature are important parameters that are widely used in the ED during procedures such as triage [7,8] and in the identification of high-risk hospital inpatients [9]. They are also recorded upon admission to the medical wards and throughout the patients’ hospital stay during almost all procedures [10]. Vital signs also reflect the patients’ current health status [11]. Thus, changing trends in patients’ vital signs may indicate clinical deterioration, which may result in adverse effects or death if not identified and treated promptly [12]. Furthermore, they can be used to determine the urgency for intensive care unit transfer [13,14].

A systemic review published in 2011 examined the evidence regarding the association of abnormal vital signs and presenting symptoms with increased mortality in the ED [7]. In addition, a large number of previous studies have examined this relationship in specific ways, especially in trauma [15,16], critically ill [17], surgically free [3,17], and ED patients [18]. Other studies were conducted in different countries such as Singapore [11], Sweden [6,17], and the United States [19]. Similarly, previous research has revealed that abnormal vital signs were strongly associated with a higher mortality rate, age, consciousness level, oxygen saturation, and Glasgow Coma Scale (GCS) score [7,11].

The present study aimed to assess the association between abnormal vital signs and mortality from June 2020 to January 2021 at the ED of King Abdulaziz University Hospital (KAUH), a tertiary center in Jeddah, Saudi Arabia.

## Materials and methods

Study design, setting, and duration

This retrospective record review study was conducted from June 2020 to January 2021 at the ED of KAUH, a tertiary center in Jeddah, Saudi Arabia.

Study participants

The medical records of 105,837 men and women who were aged above 17 years and who visited the ED between 2018 and 2020 were analyzed. Among these, 641 patients were randomly selected using a computed system and included in this study. We excluded the patients who are below 17 years old from our study.

Measurements

The following variables were collected from each patient and analyzed: age, sex, weight, height, chief complaint, date of admission, diagnosis, death, previous emergency room (ER) visits, vital signs, medical history, and length of stay. The included vital signs were classified into different levels as follows. HR was classified into the categories of resuscitation (≥130 bpm), urgent (121-130 bpm), less urgent (111-120 bpm), and not urgent (50-110 bpm). RR was classified into the categories of resuscitation (≥34 or ≤7), urgent (31-35), less urgent (26-31), and not urgent (7-25). Body temperature was classified into the categories of urgent (≥40°C or ≤32°C), less urgent (38.1°C-40°C or 32°C-34°C), and not urgent (34.1°C-38°C). Oxygen saturation (O_2_SAT) was classified into the categories of resuscitation (≤80%), urgent (80%-89%), less urgent (90%-94%), and not urgent (≥95%). BP was classified into the categories of low (systolic BP (SBP) ≤ 90 mmHg or diastolic BP (DBP) ≤ 60 mmHg), normal (SBP = 91-130 mmHg or DBP = 60-80 mmHg), and high (SBP ≥ 131 mmHg or DBP ≥ 80 mmHg) [6].

Vitals including HR, O_2_SAT, BP, and temperature was measured using Philips Medical Systems model SureSigns VS3 (Andover, MA, USA).

In the present study, we used the Canadian Triage and Acuity Scale (CTAS), which has five levels of acuity: level 1, resuscitation; level 2, emergent; level 3, urgent; level 4, less urgent; and level 5, nonurgent [20].

Ethical considerations

Ethical approval for the study was obtained from the institutional review board of the Ethics Research Committee of KAUH, Jeddah, Saudi Arabia (reference number: 287-20).

Statistical analysis

Data were analyzed using IBM SPSS Statistics version 26 (IBM Corp., Armonk, NY, USA). Categorical variables were presented as numbers and percentages, and the chi-squared (χ2) test was used to test the relationship between variables. Continuous variables were expressed as mean ± standard deviation (SD). The Mann-Whitney test was used for nonparametric variables. Multivariate logistic regression analysis was performed to assess the risk factors (independent predictors) of death among the studied patients. The odds ratio was determined at a confidence interval of 95%. Statistical significance was set at p < 0.05.

## Results

Table 1 shows the descriptive data of the included patients. The mean age was 45.66 ± 18.43 years, 69.3% were females, and 44.6% had normal weight. The mean body mass index (BMI) was 26.71 ± 6.19 kg/m^2^. Altogether, 71.1% of the patients had CTAS level 3. More than half of the patients (55.7%) had an ER stay of less than six hours, and 76.8% of the patients had a history of ER visits in the last six months. Altogether, 40.95% of the patients had a past medical history, and the most common medical conditions were hypertension (HTN) (31.3%) and diabetes mellitus (DM) (29.8%).

**Table 1:** Distribution of patients according to their age, sex, body mass index, triage level, length of stay, previous visit, medical history, and time of death (n = 641) BMI: body mass index; SD: standard deviation; CTAS: Canadian Triage and Acuity Scale; ER: emergency room; NA: not applicable; COPD: chronic obstructive pulmonary disease; DM: diabetes mellitus; CKD: chronic kidney disease; HTN: hypertension

Variable	No. (%)
Age (mean ± SD)	45.66 ± 18.43
Sex	
Male	197 (30.7)
Female	444 (69.3)
BMI categories	
Underweight (≥18.5 kg/m^2^)	20 (3.1)
Normal weight (18.5–24.9 kg/m^2^)	286 (44.6)
Overweight (25–30 kg/m^2^)	186 (29)
Obese (≤30 kg/m^2^)	149 (23.2)
BMI (mean ± SD)	26.71 ± 6.19
CTAS level	
Level 1	15 (2.3)
Level 2	169 (26.4)
Level 3	456 (71.1)
Level 4	1 (0.2)
ER length of stay	
<6 hours	357 (55.7)
6–17 hours	192 (30)
18–24 hours	43 (6.7)
>24 hours	49 (7.6)
Previous ER visit during last year	
Yes	492 (76.8)
No	149 (23.2)
Past medical history	
Yes	262 (40.9)
No/NA	379 (59.1)
Past medical history	
Cardiovascular diseases	110 (17.2)
Asthma/COPD	40 (6.2)
Dyslipidemia	24 (3.7)
DM	191 (29.8)
Liver diseases	16 (2.5)
CKD	31 (4.8)
HTN	199 (31.3)
Timing of death	
Not applicable	609 (95)
Within hours	8 (1.2)
Within days	12 (1.9)
Within weeks	10 (1.6)
Within months	2 (0.3)

Altogether, 51.3% and 55.7% of the patients had normal SBP and DBP, respectively. The mean values of SBP and DBP were 127.88 ± 27.73 and 70.05 ± 17.32 mmHg, respectively (Table 2). Furthermore, 79.4% of the patients had not urgent HR, 90.5% of the patients had not urgent RR, 90.6% had not urgent O_2_SAT, and 69.4% of the patients had not urgent body temperature.

**Table 2:** Distribution of patients according to their vital signs SBP: systolic blood pressure; DBP: diastolic blood pressure; HR: heart rate; O_2_SAT: oxygen saturation; RR: respiratory rate; SD: standard deviation

Variable	No. (%)
SBP	
Not measured (missed data)	9 (1.4)
Low blood pressure (≥90 mmHg)	21 (3.3)
Normal blood pressure (91–130 mmHg)	329 (51.3)
High blood pressure (≤131 mmHg)	282 (44)
DBP	
Not measured (missed data)	9 (1.4)
Low blood pressure (≥60 mmHg)	127 (19.8)
Normal blood pressure (60–80 mmHg)	357 (55.7)
High blood pressure (≤80 mmHg)	148 (23.1)
HR	
Not measured (missed data)	7 (1.1)
Resuscitation (≥130 bpm)	22 (3.4)
Urgent (121–130 bpm)	37 (5.8)
Less urgent (111–120 bpm)	66 (10.3)
Not urgent (50–110 bpm)	509 (79.4)
RR	
Not measured (missed data)	8 (1.2)
Resuscitation ( ≥34 or ≤7)	19 (3)
Urgent (31–35)	7 (1.1)
Less urgent (26–31)	27 (4.2)
Not urgent (7–25)	580 (90.5)
Body temperature	
Not measured (missed data)	6 (0.9)
Urgent (≥40°C or ≤32°C)	2 (0.3)
Less urgent (38.1°C–40°C or 32°C–34°C)	15 (2.3)
Not urgent (34.1°C–38°C)	618 (69.4)
O_2_SAT	
Not measured (missed data)	-1.2
Resuscitation (≤80%)	5 (0.8)
Urgent (80%–89%)	21 (3.3)
Less urgent (90%–94%)	26 (4.1)
Not urgent (≥95%)	581 (90.6)
Mean values (mean ± SD)	
SBP	127.88 ± 27.73
DBP	70.05 ± 17.32
HR	92.5 ± 23.04
Body temperature	36.23 ± 3.59
O_2_SAT	96.96 ± 11.94
RR	21.4 ± 4.38

Altogether, 5% of the patients died after the ED visit. Most of the patients had a high BMI (mean BMI: 28 kg/m^2^). The mortality rate was significantly associated with cardiovascular disease, DM, chronic kidney disease (CKD), or HTN (p ≤ 0.05). Furthermore, no significant relationship was found between death and patients’ age, sex, length of stay, and previous ER visit (p ≥ 0.05) (Table 3).

**Table 3:** Relationship between death and patients’ age, sex, body mass index, medical history, length of stay in the emergency department, and previous emergency room visit (n = 641) BMI: body mass index; SD: standard deviation; ER: emergency room; COPD: chronic obstructive pulmonary disease; DM: diabetes mellitus; CKD: chronic kidney disease; HTN: hypertension *Mann–Whitney U test

Variable	Death			
	Yes	No	χ^2^	p-value
	No. (%)	No. (%)		
Age (mean ± SD)	44.59 ± 19.85	45.71 ± 18.36	0.55*	0.58
Sex				
Male	14 (7.1)	183 (92.9)	1.83	0.175
Female	20 (4.5)	424 (95.5)		
BMI categories				
Underweight (≥18.5 kg/m^2^)	0 (0.0)	20 (100)	7.37	0.061
Normal weight (18.5–24.9 kg/m^2^)	11 (8)	275 (96.2)		
Overweight (25–30 kg/m^2^)	9 (4.8)	177 (95.5)		
Obese (≤30 kg/m^2^)	14 (9.4)	135 (90.6)		
BMI (mean ± SD)	28.79 ± 6.13	26.59 ± 6.18	2.16*	0.031
ER length of stay				
<6 hours	17 (4.8)	340 (95.2)		
6–17 hours	13 (6.9)	179 (93.2)	1.85	0.603
18–24 hours	1 (2.3)	42 (97.7)		
>24 hours	3 (6.1)	46 (93.9)		
Previous ER visit				
Yes	24 (4.9)	468 (95.1)	0.76	0.382
No	10 (6.7)	139 (93.3)		
Past medical history				
Cardiovascular diseases	11 (10)	99 (90)	5.82	0.016
Asthma/COPD	3 (7.5)	37 (92.5)	0.41	0.522
Dyslipidemia	1 (4.2)	23 (95.8)	0.06	0.8
DM	18 (9.4)	173 (90.6)	9.19	0.002
Liver diseases	2 (12.5)	14 (87.5)	1.69	0.193
CKD	9 (29)	22 (71)	36.51	<0.001
HTN	23 (11.6)	176 (88.4)	22.48	<0.001

Table 4 shows that patients with a lower mean O_2_SAT and lower mean RR had a significantly higher mortality rate (p ≤ 0.05). On the other hand, a nonsignificant relationship was observed between death and HR categorization, temperature categorization, and mean SBP or DBP (p ≥ 0.05).

**Table 4:** Relationship between death and patients’ vital signs (n = 641) SBP: systolic blood pressure; DBP: diastolic blood pressure; HR: heart rate; O_2_SAT: oxygen saturation; RR: respiratory rate; SD: standard deviation *Mann–Whitney U test

Variable	Death			
	Yes	No	χ^2^	p-value
	No. (%)	No. (%)		
HR				
Not measured (missed data)	0 (0.0)	7 (100)	1.25	0.869
Resuscitation (≥130 bpm)	1 (4.5)	21 (95.5)		
Urgent (121–130 bpm)	2 (5.4)	35 (94.6)		
Less urgent (111–120 bpm)	2 (3)	64 (97)		
Not urgent (50–110 bpm)	29 (5.7)	480 (94.3)		
Body temperature				
Not measured (missed data)	0 (0.0)	6 (100)	1.33	0.721
Urgent (≥40°C or ≤32°C)	0 (0.0)	2 (100)		
Less urgent (38.1°C–40°C or 32°C–34°C)	0 (0.0)	15 (100)		
Not urgent (34.1°C–38°C)	34 (5.5)	584 (94.5)		
Mean values				
SBP	115.14 ± 59.65	128.59 ± 24.68	0.41*	0.677
DBP	68.76 ± 16.11	70.13 ± 17.4	1.15*	0.248
HR	96.14 ± 17.93	92.3 ± 23.29	0.95*	0.34
Body temperature	36.51 ± 0.4	36.22 ± 3.69	0.42*	0.672
O_2_SAT	81.11 ± 34.43	97.56 ± 8.47	5.29*	<0.001
RR	21 ± 10.42	21.43 ± 3.79	2.16*	0.03
Glucose	4.69 ± 5.92	3.42 ± 5.58	1.99*	0.047

**Figure 1:**
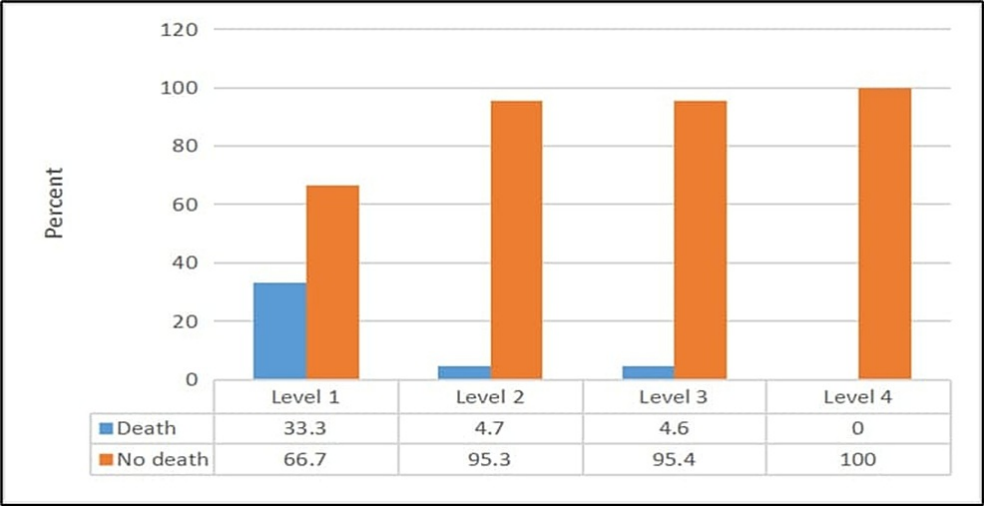
Relationship between death and Canadian Triage and Acuity Scale level χ2 = 24.07; p < 0.001

**Figure 2:**
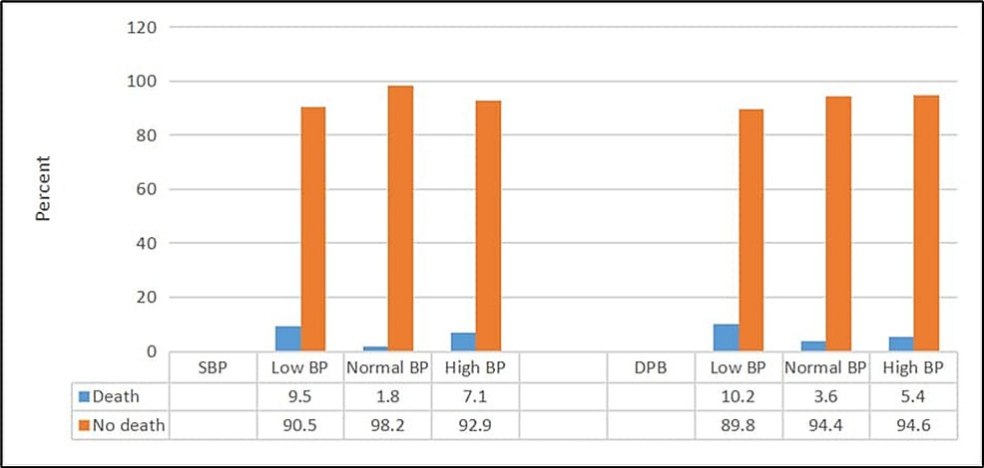
Relationship between death and systolic and diastolic blood pressures BP: blood pressure; SBP: systolic blood pressure; DBP: diastolic blood pressure For SBP: χ2 = 77.49; p < 0.001 For DBP: χ2 = 8.62; p = 0.035

**Figure 3:**
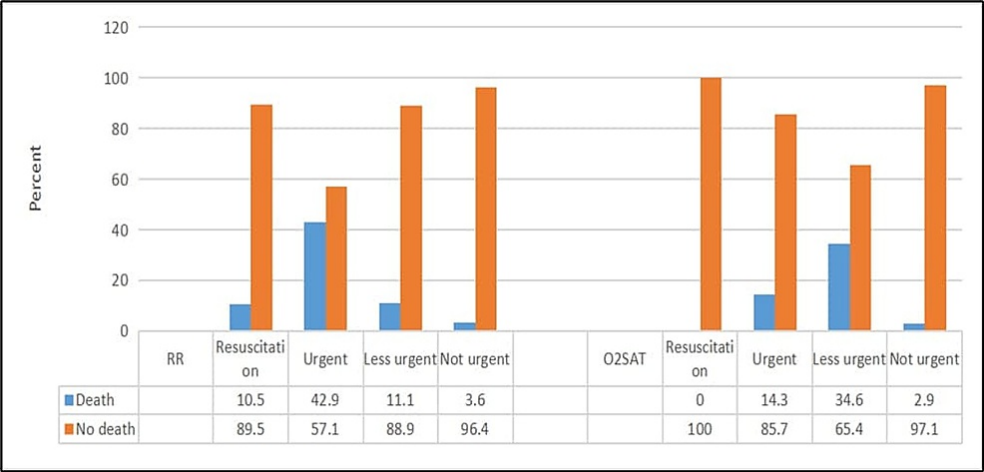
Relationship between death and respiratory rate and oxygen saturation RR: respiratory rate; O_2_SAT: oxygen saturation For RR: χ2 = 77.87; p < 0.001 For O_2_SAT: χ2 = 106.77; p < 0.001

Patients with CTAS level 1 or low SBP/DBP had a significantly higher mortality rate (p ≤ 0.05) (Figures 1, 2). Moreover, patients who had urgent RR or less urgent O_2_SAT had a significantly higher mortality rate (p ≤ 0.05) (Figure 3).

The multivariate logistic regression analysis to assess the risk factors for death revealed that CKD and less urgent O_2_SAT were significant risk factors for death (95% confidence interval: p ≤ 0.05) (Table 5).

**Table 5:** Multivariate logistic regression analysis of risk factors (independent predictors) for death (95% confidence interval) BMI: body mass index; RRCAT: respiratory rate categorization; DM: diabetes mellitus; CKD: chronic kidney disease; HTN: hypertension; SBPCAT: systolic blood pressure categorization; DBPCAT: diastolic blood pressure categorization; O_2_SATCAT: oxygen saturation categorization

Variable	B	Wald	p-value	Odds ratio (95% confidence interval)
BMI	0.04	2.16	0.142	0.95 (0.9–1.01)
RR	0.01	0.06	0.8	0.98 (0.91–0.1.07)
RRCAT	0.08	0.07	0.782	1.08 (0.6–10.94)
Glucose	0.003	0.005	0.943	0.99 (0.92–1.07)
Cardiac history	0.13	0.08	0.77	0.87 (0.33–2.24)
DM	0.54	0.102	0.312	0.57 (0.19–1.67)
CKD	1.97	13.47	<0.001	7.22 (2.51–20.78)
HTN	0.88	2.87	0.09	2.42 (0.87–6.73)
SBPCAT	0.29	0.53	0.465	1.34 (0.6–2.96)
DBPCAT	0.45	2.73	0.098	1.56 (0.92–2.67)
O_2_SATCAT	0.77	5.72	0.017	2.17 (1.15–4.11)

## Discussion

In this retrospective record review study of ED patients, we aimed to analyze the association between abnormal vital signs and mortality rate in the ED. We also aimed to identify the value of abnormal vital signs as prognostic tools for identifying patients at an increased risk of death in the hospital and enhancing the triage system.

We demonstrated that the categories of vital signs and the triage system used in our study were valid tools for predicting in-hospital mortality. Impaired RR and O_2_SAT were strongly associated with adverse outcomes. Moreover, we observed that increased BMI and a history of CKD, DM, and HTN were strongly associated with higher odds of mortality. However, the CTAS did not seem to impact mortality but helped prioritize patients with the most urgent needs.

Surprisingly, the results indicated that among the vital signs significantly associated with a higher mortality rate, greater deviation of the vital signs from their normal range was associated with lower odds of mortality. In contrast, a previous study involving a larger population showed that greater deviation of the vital signs from their normal range was associated with higher odds of mortality [21].

Respiratory rate

Low O_2_SAT [17,18,21,22] and low or high RR [3,17,21] have been identified as significant independent predictors of a high mortality rate. Our findings are consistent with the findings of these previous studies (Figure 3).

Heart rate

We observed that HR categorization was associated with decreased odds of in-hospital mortality (Figure 2). A similar conclusion was reported by other studies [3,18,21]. However, two other studies demonstrated that HR was negatively correlated with adverse outcomes [6,17,22].

A popular explanation is that HR could be affected by many environmental or internal factors, such as anxiety, noise, stress, and emotions, in addition to serious illnesses. Thus, it might contribute to inaccurate measurements and results.

Blood pressure

Interestingly, we observed that low SBP was a significant risk factor for in-hospital mortality, but high BP was not associated with a higher mortality rate. The explanation for this finding is discussed in previous studies in Switzerland [22], Sweden [21], and South Africa [3]. Other studies have failed to reveal a significant association between low SBP and higher in-hospital mortality [17,18].

Temperature

Another novel finding in the present study was that low and high body temperatures were not significant predictors of in-hospital mortality. In-hospital mortality was negatively correlated with temperature in sub-Saharan Africa [23], South Africa, and Sweden [3,17]. However, two of the seldom published studies that investigated temperature (low or high) and its relationship with mortality in the ED found contrasting outcomes [21,24,25].

It is important to highlight that the findings in these previous studies may simply reflect the abnormal temperature value distribution in the study populations (one population may be dominated by hyperpyretic patients, while the other may be dominated by hypothermic patients). A significant correlation between temperature and the in-hospital mortality rate has hardly been defined in previous studies [26].

We observed that the length of stay in the ED did not have a significant impact on the mortality rate. This finding is supported by two other studies that failed to identify the length of stay in the ED as an independent predictor of in-hospital mortality [26,27].

The present study revealed that age was not significantly associated with higher odds of one-day mortality. Similar findings were also observed in other studies in South Africa and sub-Saharan Africa [3,23]. However, previous studies in the United Kingdom and Sweden have shown that age was associated with increased mortality [6,14,17,18,28]. In these studies, the mean age of the study population was 60 years or above, while the mean age of our study population was approximately 45 years. The study populations from sub-Saharan Africa and South Africa had mean ages of 36 and 43 years, respectively.

Thus, the differences between the previous studies and our study are possibly due to the differences in the mean age of the population, since the older population is likely to exhibit higher mortality than the younger population.

Limitations

Some of the limitations of the present study include incomplete charts in the hospital’s data system and the possible inaccuracies in the chart data. Moreover, we were unable to include all patients during the study period. We selected a random sample that represented the study population.

## Conclusions

The mortality rate was significantly associated with abnormal RR, O_2_SAT, and low BP. Abnormal HR and body temperature did not show a significant association with higher mortality. HTN, DM, and CKD were also associated with higher mortality rates in patients who visited the ED.

We recommend that abnormal vital signs in the ED should receive serious and prompt attention and triage systems should be studied and reevaluated according to the characteristics of each population. Further systematic reviews and meta-analyses are needed to elucidate the association of vital signs with the mortality rate.
